# Eficácia do topiramato como terapia adicional em pacientes com estado epiléptico refratário: uma breve revisão sistemática

**DOI:** 10.5935/0103-507X.20210054

**Published:** 2021

**Authors:** Leonardo Christiaan Welling, Nícollas Nunes Rabelo, Marcia Harumy Yoshikawa, João Paulo Mota Telles, Manoel Jacobsen Teixeira, Eberval Gadelha Figueiredo

**Affiliations:** 1 Departamento de Neurocirurgia, Universidade Estadual de Ponta Grossa - Ponta Grossa (PR), Brasil.; 2 Departamento de Neurocirurgia, Universidade de São Paulo - São Paulo (SP), Brasil.

**Keywords:** Estado epiléptico, Topiramato, Convulsão

## Abstract

**Objetivo:**

Identificar evidências atuais sobre topiramato para o estado de mal epiléptico refratário.

**Métodos:**

Foi revisada a literatura para investigar a eficácia do topiramato no tratamento de estado de mal epiléptico refratário. Os termos de busca utilizados foram: “*status epilepticus*”, “*refractory*”, “*treatment*” e “*topiramate*”. Não se empregaram restrições.

**Resultados:**

A busca identificou 487 artigos que descreviam o uso de topiramato para tratamento de estado de mal epiléptico refratário e seus resultados. Relatos de caso, revisões e experimentos em animais foram excluídos. Após exclusão de duplicatas e aplicação dos critérios de inclusão e exclusão, restaram nove estudos. Realizaram-se análises descritivas e qualitativas, com os seguintes resultados: as taxas de resposta, definidas como término de crises até 72 horas após administração de topiramato, variaram entre 27% e 100%. A mortalidade variou de 5,9% a 68%. Desfechos funcionais positivos, definidos como alta hospitalar, volta à funcionalidade basal ou reabilitação, foram documentados por sete estudos, e as taxas variaram entre 4% e 55%. A maioria dos estudos reportou apenas efeitos colaterais leves ou ausentes.

**Conclusão:**

Topiramato foi efetivo em abortar estado de mal epiléptico refratário, apresentando baixa mortalidade e boa tolerabilidade. Portanto, topiramato poderia ser uma boa opção como terceira linha para estado de mal epiléptico refratário, porém mais estudos são necessários.

## INTRODUCTION

Status epilepticus (SE) is defined by the League Against Epilepsy (ILAE) Task Force as “a condition resulting either from the failure of the mechanisms responsible for seizure termination or from the initiation of mechanisms which lead to abnormally prolonged seizures”.^(1)^ It is a medical emergency associated with high mortality that demands immediate medical care and prolonged hospital stay, incurring high health care costs.^(2-6)^ The American Epilepsy Society establishes benzodiazepines as first-line treatment and fosphenytoin, valproic acid, levetiracetam, or intravenous phenobarbital as second-line.^(7)^ The state of refractory disease is characterized by the failure of first- and second-line therapies. Currently, there are few controlled or randomized studies about refractory status epilepticus (RSE) and no drug with clear evidence to be useful as a third-line treatment, so therapeutic management often includes repeating second-line therapy or anesthetic doses of either thiopental, midazolam, pentobarbital, or propofol.^(7)^

Topiramate (TPM) is being studied as an option in these refractory patients. It is a second-generation drug with an action mechanism against various epileptic syndromes with pleiotropic effects on different receptors and ion channels. Pathophysiological studies demonstrate that topiramate potentiates gamma-aminobutyric acid (GABA) through modulation of its GABAA receptor independent of benzodiazepines. This means that topiramate can help to overcome the benzodiazepine resistance observed in refractory epileptic patients.^(8)^

Given the importance of clear evidence to guide RSE therapy and the lack of studies in this area, the purpose of this systematic review is to investigate the efficacy of TPM as an add-on therapy to patients with RSE compared with those who did not use it. Addressing this question is fundamental to instruct medical conduct, improve health care, and reduce costs of treatment. We carried out a systematic review to identify current evidence on the use of topiramate for RSE.

## METHODS

To investigate the efficacy of topiramate as an add-on therapy to patients with RSE compared with those who did not use it, electronic searches were performed by two reviewers independently in March 2020 in four different databases: MEDLINE, Embase, Cochrane Library and Web of Science. The search terms were “status epilepticus”, “refractory”, “treatment,” and “topiramate”. No restrictions were used. The inclusion criteria were as follows: studies reporting the use of topiramate as a treatment for RSE and its outcomes (response rate, mortality rate, or long-term outcomes). Case reports, review articles, letters, conference abstracts and animal experiments were excluded. After the study selection, we performed descriptive and qualitative analyses. For each study, we evaluated the study design, the number of participants, the dose of topiramate administered, response rate 72 hours after the administration of TPM, the mortality rate in-hospital, and favorable long-term outcomes (i.e., discharge, back to baseline or rehabilitation). Only the data of patients who had TPM as the last drug were included.

## RESULTS

**Figure 1:**
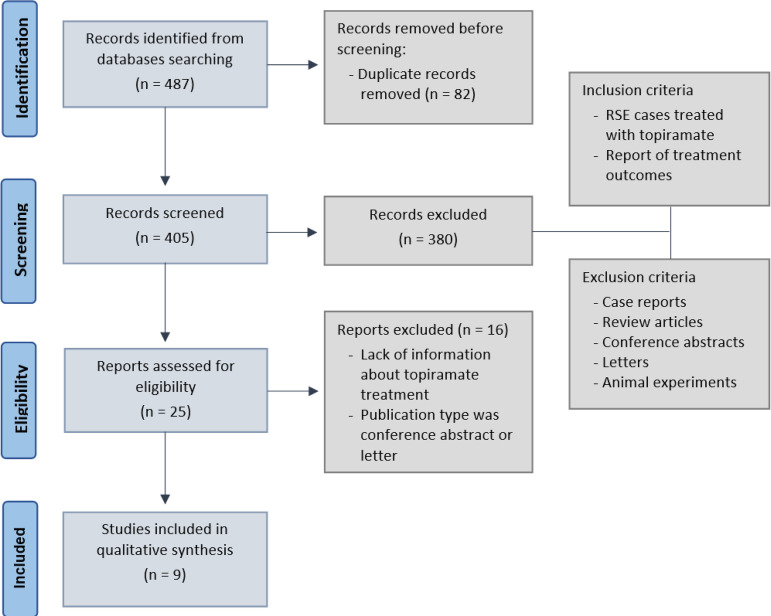
Study selection flow diagram for the systematic review. RSE - refractory status epilepticus.

The search returned 487 articles, including 82 duplicates. We screened 405 studies, resulting in 25 manuscripts eligible for full text assessment; among those, 16 studies were excluded due to lack of information about topiramate treatment outcomes and publication type. Nine studies were included in this review (Figure 1).

Eight studies were retrospective, and one was prospective.^(9)^ Madzar et al.^(10)^ was the only one that retrospectively compared episodes treated with and without TPM in terms of demographics, RSE characteristics, clinical course, and outcome; the others only analyzed cases treated with TPM. No studies were controlled or randomized. The total number of patients included was 261, with the number of participants in each study varying from 6 to 106 (Table 1).

**Table 1:** Studies included

Author	Study design	Nº of cases	Daily dose	Response* (%)	Mortality (%)	Favorable long-term outcome† (%)
Asadi-Pooya et al. ^(9)^	Prospective	20	400mg	80	35	55
Madzar et al. ^(10)^	Retrospective	17	50mg - 1,000mg	100	5.9	4
Akyildiz et al. ^(11)^	Retrospective	14	5mg/kg - 25mg/kg	85	7	21
Fechner et al. ^(12)^	Retrospective	106	100mg - 400mg	27	22.6	21.7
Synowiec et al. ^(13)^	Retrospective	27	400mg - 600mg	48	18.5	NA
Hottinger et al. ^(14)^	Retrospective	27	< 400mg - 800mg	81.4	33	66
Towne et al. ^(15)^	Retrospective	6	300mg - 1,600mg	66	NA	NA
Stojanova et al. ^(16)^	Retrospective	11	50mg - 800mg	27	36	9
Kim et al. ^(17)^	Retrospective	16	300mg - 1,000mg	81	68	25

NA - not assessed.

* Response defined as termination in hospital stay until 72 hours after the administration of topiramate; † favorable long-term outcome defined as discharge, back to baseline or rehabilitation.

There was no significant difference in the population between the studies concerning gender and age, except for one study^(11)^ that included only pediatric patients. Most studies included participants with different types of seizures, including generalized clonic, generalized tonic-clonic, simple partial, complex partial, nonconvulsive, and focal motor seizures. Fechner et al.^(12)^ did not report the type of seizure of patients, and Asadi-Pooya et al.^(9)^ only included patients with generalized convulsive status epilepticus. Synowiec et al.,^(13)^ Hottinger et al.,^(14)^ and Asadi-Pooya et al.^(9)^ documented a history of epilepsy among 45.7%, 31.4%, and 20% of patients, respectively.

Refractory status epilepticus etiology was diverse within and between studies and included infection, intracranial hemorrhage, low antiepileptic drug (AED) level, metabolic abnormality, drug or alcohol overdose or withdrawal, trauma, stroke, anoxia/hypoxia, brain tumor, congenital brain malformation, myocardial infarction, Dandy-Walker syndrome, and Lennox Gestaut syndrome.

Refractory status epilepticus severity was assessed with the Status Epilepticus Severity Score (STESS) by two studies: Madzar et al.^(10)^ and Fechner et al.^(12)^ The former reported STESS ≥ 3 in 7% of patients treated with TPM and in 36% of patients not treated with TPM; the latter reported STESS 0 - 3 in 64.2% of the patients included and STESS 4 - 6 in 35.8%.

The maximum daily dose of TPM used in each study had considerable variation, ranging between 400mg and 1,600mg, while the minimum daily dose varied from 50mg to 400mg. Even within studies, the dose administered for each patient showed remarkable variation (Table 1).

The response rates, here defined as termination in-hospital until 72 hours after the administration of TPM, varied from 27% to 100%. The mortality rate varied from 5.9% to 68%. One study^(15)^ did not report the mortality rate. Positive functional long-term outcome - defined as discharge, back to baseline, or rehabilitation - was documented by seven studies, and the rates ranged between 4% and 55%. The study performed with pediatric patients reported 21% discharge without neurological sequelae in the follow-up.

Most studies reported no or slight adverse effects that involved metabolic acidosis, hyperammonemia, later nephrolithiasis (occurring in one patient 63 days after TPM introduction and leading to sepsis), and lethargy. However, Fechner et al.^(12)^ observed a significant rate of hyperammonemia during treatment with TPM - 35.8% of the patients developed that disturbance.

## DISCUSSION

Topiramate demonstrated response rates similar or even superior to those documented by the current third-line options to RSE (pentobarbital 4% - 43%, propofol 46% - 62%, or midazolam 63% - 100%).^(18,19)^ Moreover, a study that compared episodes treated with and without TPM^(10)^ reported that the likelihood of RSE termination was significantly higher when TPM was part of the baseline AED regimen.

Intriguingly, in studies with more significant variability in TPM doses,^(15,16)^ lower doses seem to be associated with higher response rates. However, the heterogeneous RSE etiologies and TPM cotherapy are significant biases that disallow the association of lower doses with higher response rates. Concerning etiologies, previous studies demonstrated that epilepsy and previous diagnosis of epilepsy offer a favorable prognosis, while coma and RSE caused by anoxia/hypoxia were unfavorable factors.^(19-23)^

Mortality seems to be lower than that observed in other antiepileptic drugs,^(19,21)^ which could be associated with the characteristics of the patients chosen to receive TPM therapy. Madzar et al.^(10)^ documented that TPM seemed to be administered to younger and healthier patients in association with higher doses of AEDs. It is essential to note that younger age alone is not a predictor of better outcomes in RSE, but the worse clinical course of older patients is most strongly correlated with underlying etiologies and comorbidities.^(19,21-26)^

The study’s significant limitations were the heterogeneity of the population studied (i.e., the varying etiologies and severity levels of RSE, variance in the protocol of administration of TPM, and the use of different doses and cotherapies). Most importantly, these limitations demonstrate the lack of high-quality evidence on this topic, particularly in comparing topiramate to other treatments for RSE.

Despite these limitations, our study demonstrates the likely efficacy of TPM in RSE episodes and the necessity of large, controlled, and randomized trials that could provide clear evidence. Furthermore, the formulation of intravenous solutions of TPM is essential to increase its use in situations of SE, although oral TPM has good bioavailability, little protein binding, and rapid absorption.^(27)^ Fortunately, intravenous solutions are under development for clinical practice.^(28)^

## CONCLUSION

Topiramate was effective in terminating refractory status epilepticus. Its response rate seems similar or even superior to those documented by the current third-line options for refractory status epilepticus, while mortality seems lower. Despite the difficulty of evaluating adverse events associated with add-on medications in critically ill patients, topiramate was well tolerated and promoted no severe side effects, so it can be considered a good option as third-line therapy for refractory status epilepticus. Further studies are needed to directly compare topiramate with other currently recommended drugs.
